# Social engineering of societal knowledge in livestock science: Can we be more empathetic?

**DOI:** 10.14202/vetworld.2017.86-91

**Published:** 2017-01-23

**Authors:** R. K. Ravikumar, Devesh Thakur, Hardev Choudhary, Vivek Kumar, Amol S. Kinhekar, Tushar Garg, K. Ponnusamy, G. R. Bhojne, Vasanth M. Shetty, Vipin Kumar

**Affiliations:** 1 National Innovation Foundation-India, Satellite Complex, Ahmedabad 380 015 Gujarat, India; 2 Department of Veterinary and Animal Husbandry Extension Education, Dr. GC Negi College of Veterinary and Animal Sciences, CSK Himachal Pradesh Krishi Vishvavidyalaya, Palampur - 176 062, Himachal Pradesh, India; 3 Division of Dairy Extension, ICAR- National Dairy Research Institute, Karnal - 132 001, Haryana, India; 4 Department of Clinical Medicine, Ethics & Jurisprudence, Nagpur Veterinary College, Maharashtra Animal & Fishery Sciences University, Nagpur - 440 001, Maharashtra, India; 5 Dean, Veterinary College, Karnataka Veterinary, Animal and Fisheries Sciences University, Hassan - 573 202, Karnataka, India

**Keywords:** health, indigenous knowledge, innovation, institution, livestock, technology

## Abstract

Questions are raised in effective utilization of farmer’s wisdom by communities in their farming. Planners support to livelihood emphasize mostly of inputs from outside and not setting up sustainable goals. Formal institutions and planners of program are finding constraints and sceptical in wider dissemination of indigenous knowledge research system (IKRS). This is in spite of evidence that considerable number of farmer’s in livestock sector depends on IKRS. In this context, it is pertinent to showcase dissemination potential of these knowledge system(s) in larger geographical areas. The review illustrates different challenges encountered while control of livestock ailments like ectoparasite infestation through IKRS. Several times, it was opinioned to provide or share IKRS to thwart ailments in a specific region. This is interesting as it was narrated how formal system is unable to recognize farmer’s problem and challenges in integrating these sustainable practices. It has to be noted that disseminating activities seldom takes into account the experimental potential of farmers. This review paper articulates various evidences generated in enhancing diffusion thereby dissemination of IKRS. The nature of support extended by IKRS in entrepreneurial activity of smallholder farming units did not get adequate recognition. There needs to be minimum standard protocol in deriving benefit from such low-cost alternative technologies. This will enrich incremental innovation activities as per location specific need and provide scope for wider dissemination.

## Introduction

### Technology, farmer’s insight, and civil society

Extent of adoption and utilization of technology depends on access to financial resources [1] and resource endowment of farmers [2]. The upfront cost of investment creates hurdle for households in adoption of technologies [3]. Concern is expressed in development, generation of knowledge base in quickest possible time to meet problems in agriculture [4]. It is reiterated that farmers through their own observations continue to develop and apply new technologies [5]. Farmers engage in different social roles [6], and there is a need to scale up such process for sustaining public health. Functional association among actors, social assets, local institutional care, ease of use of available resources, ability to take risk determine intensification of technological utility, which may demand different transfer approach [7]. In this process, social network plays key determinant [8]. Concerted efforts need to be oriented toward technology demonstration and social engagement among women for enhancing farming standards [9]. Practices derived from indigenous system have the main role in farming engaged by resource-poor farmers [10]. These knowledge systems can complement formal system in controlling livestock ailments [11] and provide solutions where accessibility is constraint [12]. Despite huge published materials on indigenous technical knowledge, in most regions integrating and effective use of this knowledge system remains elusive. The veterinary service is yet to gear up for investigating problems of farmers or mechanism to determine undifferentiated complications [13]. In this context, technologies are visualized as valuable tool to ensure food security and lessons have to be learnt in improvised use for livestock health [14]. Modeling of technology by society is crucial for diffusion at farmer’s field [15]. The framework for livestock service needs to be compatible with prevailing farming system [16]. It is vital to forge partnership among formal and informal institutions for developing meaningful technologies [17].

### Planners program, need for innovations

Technologies tailored to match with socio-cultural identity can ensure minimum disconnect with planners and actors in action [18]. The nature of advisory support extended by institutions in farmers premise influence implementation activities and to meet local requirements [19]. It has to be noted that technologies and institutional working arrangements determine governance structure of farmer’s entity [20]. Studies had observed that planners envisage farmer’s as receiver and unable to find ways to imbibe experimental nature of people [21]. Government initiated tick control program had faced difficulties as farmers faced lack of supply of acaricides [22]. It is referred that limited technologies developed from national agricultural research system were being utilized by farmers [23]. Farming activities have to look into concept of social acceptability apart from factors like existing amenities [24]. Farmers attitude toward medications play a key role in disease control strategies [25]. Their effective participation determines success of technology transfer [26]. Actor specific comprehensions are needed for ensuing collaborative governance with minimal regulation and external interventions [27].

The need to facilitate innovation as instrument of change in agriculture has been felt to ensure participation of farmers in research, value chain [28]. The critical component in implementation of innovation is to understand socio-environmental settings for benefit of communities [29]. Experience in scaling up of innovation in agricultural research suggested that farmers need to influence as an actor and recipient of it [30]. Their network lends credentials to innovations and diffusion of technical knowhow. Farmers related each other with collective processes of innovation framework were found better equipped than farmers who relied on expert support [31]. Opportunity structure for scaling up of such innovations have to be evaluated which might involve institutional change [32]. The risk taking ability or attitude of innovators is critical for advancement, research, and development [17]. There were also instances that innovation attributes of technologies shared by formal units as limiting factors. It was also noticed that less validation of technologies in farmer’s field had led to limited adoption of dairy innovations [33].

### Distributed innovation model and indigenous knowledge research system (IKRS)

In design elements of distributed innovation system individuals work in independent entity but part of technological system [34]. In this system, knowledge socialization have to occur so as to promote an environment to transform and benefit from creativity [35]. The critical contest is to access this knowledge which is generally outside institutional boundaries [36]. Antibiotic residues, indiscriminate use along with heavy economic loss in ailments like subclinical mastitis calls for different approaches in control of such ailments [37]. Livestock keepers were not equipped to meet cost of drugs in cattle health management [38]. Advancement of disease control strategies in developing countries could not translate research findings into action and failed to recognize inherent constraints [39]. In most developing countries, plant-based primary health plays an important role in livestock health system [40,41]. These IKRS sustained by outstanding knowledge holders were widely utilized in India, and there is need to strengthen it [42]. This is relevant as ecotoxicity of veterinary drugs in treatment of tick infestation has been articulated [43]. Strategies were sought to develop a program that is precise to each farm and monitored at local level in specific reference to parasite control in livestock [44]. Technology adoption depends on knowledge of people, support service, and meeting local requirements [19]. Henceforth, tailor made technological alternatives are in need to address difficulties in farming system [45].

### Applicability of knowledge system(s) and pathways of IKRS implementation

Concerted efforts to utilize indigenous knowledge system by keeping knowledge holders in forefront have been limited. Honey Bee Network globally articulated the need to link people to people, relevance of intrinsic motivation and participation of informal society through their experimentation spirit in sustaining, benefiting from knowledge systems. National Innovation Foundation (NIF), India tried different approaches in enhancing such scope of interface among creative communities and farming community. Technologies were scientifically validated with help of farmers in regions - such as Amarapur (Gandhinagar district, Gujarat), Bir (Himachal Pradesh) [46], Hadida (Amreli district, Gujarat) [11], Pondicherry (Pondicherry UT), Vanki (Dang district, Gujarat) [12], and Yerangoan (Maharashtra) [42] - State Veterinary Universities, agricultural universities had provided their valuable support in validating claims of outstanding knowledge holders toward treatment of infectious ailments and metabolic disorders [47]. This indigenous knowledge system can complement poultry farming system [48]. Attempts were undertaken primarily to know the relevance of this technical knowhow and possibility to fill gap in social settings. Further, studies conducted by NIF-India illustrated examples of indigenous knowledge practices which can work elsewhere and not necessarily at source of knowledge. This had proved that IKRS need not be with innovation characteristics of stickiness. This technical knowhow can cross geographical, cultural barriers to minimize livestock health concerns.

These are highly relevant as technologies validated against bloat were proved effective in minimizing greenhouse gas production [49]. Traditional knowledge holders, *viz*., Periyaveeturaman (Tamil Nadu), Shatadal Ghorai, Narugopal Ghorai (West Bengal), S Munda (Jharkhand), Ukhardiyabhai Raot (Gujarat), Omavati Rathore, and Geeta Rathore (Madhya Pradesh) had shared this wisdom and provided method of administration to protect livestock welfare. Traditional healers like Harshadbhai Patel (Bochasan, Anand district, Gujarat) had shown exemplary understanding of ailments such as mastitis and helped in networking among needy farmers [50]. Knowledge holders in Bishnupur district, Manipur had demonstrated capability to organize [51]. An interaction with farming community along with knowledge holder Becharbhai Devgania while sharing value addition to their knowledge proved importance of informal system by farmers [11]. This is an interface wherein knowledge system operates between creative communities, farmers, service providers to forge strong collaboration among them [6]. Understanding such attributes will enable planners to visualize and to optimally utilize these systems. Minimal support to link with different stakeholders enable refinement of technologies, development of suitable intervention thereby adoption [52]. These technologies which can meet requirements of people with low purchasing power and serve markets of low income can be treated as frugal innovations [53]. These outstanding knowledge holders enable setting up of inclusive innovation platform, which is viewed to support marginalized poor [54]. However adequate compensation to custodian of knowledge, society remains a challenge [21].

### Operationalization of IKRS through open source innovation system (OSIS): Evidence from the university research system

Globally, indigenous knowledge systems have adequately demonstrated efficacy against tick infestation and welfare of livestock. These medications were known with minimal development of resistance and adding significant value in public health. In India, practices comprising either or both of Neem (*Azadirachta indica A Juss*), Nagod (*Vitex negundo L*.) were widely known among farming communities in controlling ticks as well as other medicinal properties. However, scanty literature is available in reinforcing these knowledge systems with society. NIF-India, felt an urgent need to control tick infestation owing to farm animal welfare, emotional difficulties of farmers, public health, lack of availability of alternative options apart from reviving knowledge systems [45]. Apart from carriers of infectious agents, ectoparasitic infestation causes blood loss among livestock [55]. These ticks cannot be completely eradicated and strategies have to be oriented toward managing farm animal health locally [56]. Efficacy trials were conducted based on such knowledge in village Amarapur, Gandhinagar district, Gujarat, and found synergistic effect of combined crude extract of neem and nagod. The study found that in <48 h duration of treatment, 100% efficacy was observed [57]. A national campaign was launched by NIF to control tick infestation using OSIS approach [58]. Sharing successful findings enabled collaborative effort with Dr. GC Negi, College of Veterinary and Animal Sciences, Palampur, Himachal Pradesh. University felt the importance of such sustainable practices in hilly areas and leveraged demonstration of technical-knowhow in different regions of Bhawarna, Bir in Kangra district, Himachal Pradesh [46]. The technology transfer model had showcased evidence where farmer’s problem was addressed through their own knowledge system with help of interface among research and line departments. Immediate intervention in minimizing difficulties of farmers by veterinary institutions would farm activities [59]. The approach reiterates the principle that mainstreaming this knowledge system requires favorable atmosphere which had focused on human relationship [60]. Demonstrations of these technical know-how were held across the country, *viz*., Gujarat, Himachal Pradesh, Andhra Pradesh, and Maharashtra [46,61,62].

### IKRS and Socially Desirable Products

Several approaches were tried of which *in-situ* *incubation model of alternative technologies* (IMAT) was found to be effective to reduce gap between formal institutions and primary stakeholders [62]. Collective insight and sensitivity of stakeholders like farmers, resource personnel from formal scientific or service delivery institutions have to be part of implementation program for protecting livestock welfare. Acquaintance of farmers and their social networking skills have to be harnessed for effective livestock service. Community oriented research approach would ease the task of accessing value added knowledge systems. Technologies that were open sourced may be dovetailed into IMAT model. This can augment local actions that are sustainable and meet green challenge [63]. In case of novel medications, the development of technologies share a pathway of nonlinear innovation system [51,58]. Organized measures have to be enhanced for appreciating local innovations toward development of unique technologies. Linear approach of origin of technologies from formal research system can be complemented through such niche specific indigenous knowledge systems. Leveraging experimental wisdom of society requires construction of meaningful situation where communities meet, seek research questions with knowledge holders. These natural experimenters through their expertise acted as “gatekeeper” of information to derive affordable excellence. This requires an institutional support to cater the novel technologies shared by outstanding community or knowledge holders. The frequent use of medications in veterinary science and in large region, scale necessitated to conduct environmental analysis for products [64]. Studies had shown that IKRS through socially relevant research facilitate development of socially acceptable products [12]. These rural innovations or technologies have positive impact in livelihood of communities [65]. It was found that such products had ensured farmers to save cost of 22.875 USD per animal toward treatment of mastitis [49]. These innovations or technologies at smallholder society have to be integrated instead of treating them as separate entity to overcome farm level constraints [66]. It can help livestock institutions to experience direct experimentation by designing set of protocols with help of farmers and enable scale up of creative solutions [67]. Several technologies of outstanding knowledge holders were either validated through network with farmers, dairy cooperative societies, animal husbandry departments or through university research systems by NIF-India [47]. Technology demonstration held in regions of Pune district, Maharashtra with help of Pune Zilha Sahakari Dudh Utpadak Sangh Maryadit (Katraj Dairy) enabled farming community to recognize natural resources in their vicinity in terms of medicinal property to control tick infestation. Validated results along with method of preparation were shared at their farm. Thus, large scale adoption of this technical know-how based on established knowledge system has been translated into desirable action. Similarly technology sustained by an outstanding knowledge holder Periyaveeturaman, Tamil Nadu was sought by farmers in Gandhinagar district of Gujarat. Acaricide resistance and use of synthetic compounds created emotional difficulties to farmers as well as environmental concern. Hence, repudiation of this knowledge from distant location was not the perception as farmers were devoid of choice. Farmer led innovation program along with setting up of social assets have to be reinforced through suitable policy intervention. Scientifically validated technologies against ailments such as mastitis, ephemeral fever, tick, retention of placenta, worm infestation, bloat, respiratory distress (bird), coccidiosis (bird), and feed supplements have to be utilized. Social engineering in local manufacturing and development of these herbal products would facilitate the upscaling. Regulatory mechanisms need to evaluate risk assessment apart from other parameters in enforcing availability of products [68]. This regulatory compliance for herbal products differs in countries and regions [69]. Harmonized standards would further help to maintain uniform safety and efficacy in herbal medicines through science based approach [70] for sustainable and eco-friendly livestock farming.

## Conclusion

Livestock Wealth in India has been widely distributed and efforts are made by the public system by setting-up functional units near to farming activities. Nature of livestock rearing, smallholding units, labor intensive activities, mobility of veterinary service delivery usually poses greater challenges in sustaining livestock health. Since the feedback loop and flow of information between, within veterinary units is increasingly difficult, expanding of quality service needs to relooked. Lack of effective communication and less transfer of knowledge within community resulted in strain on livestock health service at specialized veterinary units. Dramatized problems were attended due to lack of resource, time and orientation of livestock service system. Operating practices at farmer’s field need to take measures in identifying IKRS through feedback system in line departments, viz., animal husbandry department. Technological adoption of indigenous practices and demonstration of no or low cost sustainable practices can make veterinary units functionally more effective. Regulatory guidelines need to be evaluated and updated so as to facilitate use of novel practices thereby mainstreaming of indigenous wisdom. The review reiterates strengthening of linkage among these knowledge systems and end users for wider social benefit.

## Authors’ Contributions

All authors participated in the discussion, drafting and revision of the manuscript. RK, DT, HC, VKr, ASK, GRB, VMS had carried out investigations and experimentation’s on the review topic. RK, DT, HC, ASK enabled appropriate literature for developing the manuscript. RK, DT, TG, KP, VMS, VK corrected errors, internalized and designed the content of manuscript. All authors read and approved the final manuscript.

## References

[1] Abate G.T, Rashid S, Borzaga C, Getnet K (2016). Rural finance and agricultural technology adoption in Ethiopia: Does the institutional design of lending organizations matter?. World Dev.

[2] Grabowski P.P, Kerr J.M, Haggblade S, Kabwe S (2016). Determinants of adoption and disadoption of minimum tillage by cotton farmers in eastern Zambia. Agric. Ecosyst. Environ.

[3] Bensch G, Grimm M, Peters J (2015). Why do households forego high returns from technology adoption? Evidence from improved cooking stoves in Burkina Faso. J. Econ. Behav. Organ.

[4] Ibrahim M.A.R, Dorina M, Abdelrazek I (2014). How rural agricultural development projects (animal production) can use projects benefits for improving the economics states of farmers. Procedia Econ. Finance.

[5] Cock J, Oberthür T, Isaacs C, Läderach P.R, Palma A, Carbonell J, Victoria J, Watts G, Amaya A, Collet L, Lema G, Anderson E (2011). Crop management based on field observations: Case studies in sugarcane and coffee. Agric. Syst.

[6] Hauser M, Lindtner M, Prehsler S, Probst L (2016). Farmer participatory research: Why extension workers should understand and facilitate farmers role transitions. J. Rural Stud.

[7] Birhanu M.Y, Girma A, Puskur R Determinants of success and intensity of livestock feed technologies use in Ethiopia: Evidence from a positive deviance perspective. Technol. Forecast Soc. Change, In Press. (23 September 2016).

[8] Magnan N, Spielman D.J, Lybbert T.J, Gulati K (2015). Leveling with friends: Social networks and Indian farmers demand for a technology with heterogeneous benefits. J. Dev. Econ.

[9] Sharma P, Ponnusamy K, Kale R.B (2014). Study on behavioural changes among women SHGs and their impact on adoption of scientific practices in dairying. Indian J. Anim. Res.

[10] Ponnusamy K, Kale R.B, Ravi K.N, Arulmozhi D.M.C, Sharma P (2015). Cross-regional analysis on usage of indigenous technical knowledge in dairy farming. Indian J. Anim. Res.

[11] Devgania B.S, Khordia D, Chodvadiya M.B, Patel R, Patel D, Kinhekar A.S, Singh P.K, Kumar V, Bhojne G.R, Ravikumar R.K, Kumar V (2015). Reverence of community towards grassroot livestock innovation: Responding to stakeholders need against sub-clinical mastitis in Amreli District, Gujarat, India. Adv. Anim. Vet. Sci.

[12] Raot U.S, Parmar M, Patel P, Patel R, Gogoi D.M, Patel J, Sivapregasam V, Kumar V, Ravikumar R.K, Kumar V (2016). Value addition of novel herbal livestock medication mastiherb in treatment of mastitis sustained by creative communities from the regions of Dang, Gujarat. Int. J. Bioresour. Stress Manage.

[13] Chander M, Dutt T, Ravikumar R.K, Subrahmanyeswari B (2010). Livestock technology transfer service in India: A review. Indian J. Anim. Sci.

[14] Fitzpatrick J.L (2013). Global food security: The impact of veterinary parasites and parasitologists. Vet. Parasitol.

[15] Ndyabawe K, Kisaalita W.S (2014). Diffusion of an evaporative cooler innovation among smallholder dairy farmers of Western Uganda. Technol. Soc.

[16] Rangnekar D.V (2006). Livestock in the Livelihoods of the Underprivileged Communities in India: A Review.

[17] Gupta A.K (2016). Grassroots Innovation: Minds on the Margin Are Not Marginal Minds.

[18] Warren C.R, Burton R, Buchanan O, Birnie R.V (2016). Limited adoption of short rotation coppice: The role of farmers socio-cultural identity in influencing practice. J. Rural Stud.

[19] Zander K.K, Mwacharo J.M, Drucker A.G, Garnett S.T (2013). Constraints to effective adoption of innovative livestock production technologies in the Rift Valley (Kenya). J. Arid Environ.

[20] Huang Z.H, Wu B, Xu Z.C, Liang Q (2016). Situation features and governance structure of farmer cooperatives in China: Does initial situation matter?. Soc. Sci. J.

[21] Rathore O, Rathore G, Patel C, Kinhekar A.S, Mangwani N, Kumar V, Ravikumar R.K, Kumar V (2016). Clinical evaluation of an indigenous practice against subclinical mastitis. Rumin. Sci.

[22] Sungirai M, Moyo D.Z, Clercq P.D, Madder M (2016). Communal farmers perceptions of tick-borne diseases affecting cattle and investigation of tick control methods practiced in Zimbabwe. Ticks Tick Borne Dis.

[23] Ponnusamy K, Gupta J, Nagarajan R (2009). Indigenous technical knowledge (ITK) in dairy enterprise in coastal Tamil Nadu. Indian J. Tradit. Knowl.

[24] Marzban S, Allahyari M.S, Damalas C.A (2016). Exploring farmers orientation towards multifunctional agriculture: Insights from northern Iran. Land Use Policy.

[25] Velde F.V, Claerebout E, Cauberghe V, Hudders L, Van Loo H, Vercruysse J, Charlier J (2015). Diagnosis before treatment: Identifying dairy farmers determinants for the adoption of sustainable practices in gastrointestinal nematode control. Vet. Parasitol.

[26] Ponnusamy K, Ambasankar K (2006). Technological interventions for socio-economic enrichment of dairy farmers. Indian J. Dairy Sci.

[27] de Loë R.C, Murray D, Simpson H.C (2015). Farmer perspectives on collaborative approaches to governance for water. J. Rural Stud.

[28] Ton G, Klerkx L, Grip K.D, Rau M.L (2015). Innovation grants to smallholder farmers: Revisiting the key assumptions in the impact pathways. Food Policy.

[29] Schindler J, Graef F, König H.J (2016). Participatory impact assessment: Bridging the gap between scientists theory and farmers practice. Agric. Syst.

[30] Bellotti B, Rochecouste J.F (2014). The development of conservation agriculture in australia-farmers as innovators. Int. Soil Water Conserv. Res.

[31] Dolinska A, D’aquino P (2016). Farmers as agents in innovation systems. Empowering farmers for innovation through communities of practice. Agric. Syst.

[32] Roling N (2009). Pathways for impact: Scientists different perspectives on agricultural innovation. Int. Agric. Sustain.

[33] Thirunavukkarasu D, Narmatha N (2016). Lab to land-factors driving adoption of dairy farming innovations among Indian farmers. Curr. Sci.

[34] Baldwin C.Y Organization Design for Distributed Innovation, Working Paper, 12-100, Harvard Business School.

[35] Sawhney M, Prandelli E (2000). Communities of creation: Managing distributed innovation in turbulent markets. Calif. Manage. Rev.

[36] Lakhani K.R, Panetta J.A (2007). The principles of distributed innovation. Innov. Technol. Gov. Glob.

[37] Bhikane A.U, Hase P.B, Syed A.M, Ghoke S.S, Devangare A.A, Awandkar S.P (2011). Efficacy of herbomineral formulation in subclinical mastitis in crossbred cows. Indian Vet. J.

[38] Siegmund-Schultze M, Lange F, Schneiderat U, Steinbach J (2012). Performance, management and objectives of cattle farming on communal ranges in Namibia. J. Arid Environ.

[39] Rich K.M, Perry B.D (2011). The economic and poverty impacts of animal diseases in developing countries: New roles, new demands for economics and epidemiology. Prev. Vet. Med.

[40] Raza M.A, Younas M, Buerkert A, Schlecht E (2014). Ethno-botanical remedies used by pastoralists for the treatment of livestock diseases in Cholistan desert, Pakistan. J. Ethnopharmacol.

[41] Joshi D.D (1991). Traditional Veterinary Medicine in Nepal, Animal Production and Health Commission for Asia and the Pacific (APHCA) Information Exchange Unit, No. 10.

[42] Ghorai S, Ghorai N, Dutta L, Bera A, Ghorui S, Kinhekar A.S, Ingle V.C, Sonkusale P, Awandkar S.P, Tembhurne P.A, Kumar V, Ravikumar R.K, Kumar V (2016). Protective and immuno-modulatory effect of low cost locally available technology from West Bengal, India under indigenous knowledge research system. J. Immunol. Immunopathol.

[43] Beynon S.A (2012). Potential environmental consequences of administration of ectoparasiticides to sheep. Vet. Parasitol.

[44] Taylor M.A (2013). Parasite control in sheep: A risky business. Small Rumin. Res.

[45] Kadivendi M, Maheswari R, Ravikumar R.K, Chauhan M.M, Kinhekar A.S, Kumar V, Kumar V (2015). Integrated approach for engaging farming community - Opportunities and challenges for low cost inputs. Int. J. Agric. Innov. Res.

[46] Thakur D, Sharma A.K, Ravikumar R.K, Kumar V (2016). Benefit to end users: Appraisal of extending technology at farm fields from regions of Himachal Pradesh, India. J. Exp. Biol. Agric. Sci.

[47] Kumar V, Ravikumar R.K (2016). Realistic aspiration for livestock health care through indigenous veterinary system in India, Dairy of India, 216-17.

[48] Ravikumar R.K, Kinhekar A.S, Ingle V.C, Sonkusale P, Awandkar S.P, Tembhurne P.A, Kumar V (2016). Effect of heat stress on haematological and immunological parameters in broiler chicken. Indian J. Vet. Sci. Biotechnol.

[49] Munda S, Pandey R, Bhojne G.R, Dakshinkar N.P, Kinhekar A.S, Kumar V, Ravikumar R.K, Kumar V (2016). Indigenous knowledge research system [IKRS] for treatment of bloat and its significance towards greenhouse gas emission: Jharkhand, India. Adv. Anim. Vet. Sci.

[50] Ravikumar R.K, Petharajanna B, Govindan N, Vivekanandan P, Alagumalai V, Subramanium Y, Patel J, Patel H, Hariharan M, Kumar V, Kumar V (2016). Science, society and humanity in mainstreaming indigenous knowledge research system (IKRS) from southern regions of India: An evidence for honey bee network (HBN) philosophy. Adv. Anim. Vet. Sci.

[51] Ravikumar R.K, Kumar V, Khuman L.Y, Kinhekar A.S, Thakur D, Kumar V (2016). Integrating indigenous knowledge research system (IKRS) and/with livestock health intervention program to complement natural resource conservation. Adv. Anim. Vet. Sci.

[52] Choudhary H, Ravikumar R.K, Kumar V (2016). Assessing behaviour of farmers in linking to village institution: Dairy society’s perspective from semiarid regions, Gujarat, India. J. Exp. Biol. Agric. Sci.

[53] Simula H, Hossain M, Halme M (2015). Frugal and reverse innovations - Quo Vadis?. Curr. Sci.

[54] Swaans K, Boogaard B, Bendapudi R, Taye H, Hendrickx S, Klerkx L (2014). Operationalizing inclusive innovation: Lessons from innovation platforms in livestock value chains in India and Mozambique. Innov. Dev.

[55] Singh J, Gupta S.K, Singh R, Hussain S.A (2014). Etiology and haemato-biochemical alterations in cattle of Jammu suffering from anaemia. Vet. World.

[56] Wall R (2007). Ectoparasites: Future challenges in a changing world. Vet. Parasitol.

[57] Ravikumar R.K, Kumar V, Choudhary H, Kinhekar A.S, Kumar V (2015). Efficacy of indigenous polyherbal ectoparasiticide formulation against hard tick infestation in cattle (*Bos indicus*). Rumin. Sci.

[58] Ravikumar R.K, Kumar V, Singh J, Verma H.K, Singh N, Singh S, Kasrija R (2015). New frontiers for indigenous knowledge research system [IKRS]: Non linear innovation system [NLIS] and open source innovation system [OSIS]. Lead Paper: Technologies and Proven Practices for Sustainable Livestock Production, Push to the Livestock Farming through Knowledge Empowerment of the Farmers, First National Conference, SVAHE, Guru Angad Dev Veterinary and Animal Sciences University [GADVASU], Ludhiana, India, 18-20 November.

[59] Athilakshmy S, Rao S.V.N (2013). Rearing of day old Swarnadhara chicks by farmers in Karaikal - Evidence from an action research project. Indian J. Poult. Sci.

[60] Hagmann J, Chuma E, Connolly M, Murwira K (1998). Agricultural Extension: An action Learning Experience from Zimbabwe, Network Paper, 78. Agricultural Research and Extension Network.

[61] Kumar V, Ravikumar R.K, Reddy S.K, Prasad R.M, Rao A.K (2016). Indigenous innovations in livestock production systems: NIF initiatives. Invited Papers: Innovative Designs and Implements for Global Environment and Entrepreneurial Needs Optimizing Utilitarian Sources, Indigenous, International Livestock Conference and Expo, 23^rd^ Annual Convention, ISAPM, Hyderabad, India, 28-31 January.

[62] Dhamale M, Mahajan A, Kinhekar A.S, Rajurkar G, Ravikumar R.K, Ksheersagar V.H, Kumar V (2016). Reviving technology demonstration in farmers field - An appraisal. J. Exp. Biol. Agric. Sci.

[63] Andreopoulou Z, Tsekouropoulos G, Theodoridis A, Samathrakis V, Batzios C (2014). Consulting for sustainable development, information technologies adoption, marketing and entrepreneurship issues in livestock farms. Procedia. Econ. Finance.

[64] Koschorreck J, Koch C, Rönnefahrt I (2002). Environmental risk assessment of veterinary medicinal products in the EU-a regulatory perspective. Toxicol. Lett.

[65] Yadav V, Goyal P (2015). User innovation and entrepreneurship: Case studies from rural India. J. Innov. Entrep.

[66] Ayele S, Duncan A, Larbi A, Khanh T.T (2012). Enhancing innovation in livestock value chains through networks: Lessons from fodder innovation case studies in developing countries. Sci. Public Policy.

[67] Ravikumar R.K, Periyaveeturaman C, Selvaraju D, Kinhekar A.S, Dutta L, Kumar V (2016). Community oriented ectoparasite intervention system: Concepts for on-farm application of indigenous veterinary medication. Adv. Anim. Vet. Sci.

[68] Elmgren L, Li X, Wilson C, Ball R, Wang J, Cichutek K, Pfleiderer M, Kato A, Cavaleri M, Southern J, Jivapaisarnpong T, Minor P, Griffiths E, Sohn Y, Wood D (2013). A global regulatory science agenda for vaccines. Vaccine.

[69] Fan T.P, Deal G, Koo H.L, Rees D, Sun H, Chen S, Dou J.H, Makarov V.G, Pozharitskaya O.N, Shikov A.N, Kim Y.S, Huang Y.T, Chang Y.S, Jia W, Dias A, Wong V.C, Chan K (2012). Future development of global regulations of Chinese herbal products. J. Ethnopharmacol.

[70] Knoss W (2015). Harmonization of regulatory requirements in Europe to ensure quality, safety and efficacy of herbal medicinal products. Evidence-Based Validation of Herbal Medicine.

